# Analysis of Key Chemical Components in Aqueous Extract Sediments of Panax Ginseng at Different Ages

**DOI:** 10.3390/foods11081161

**Published:** 2022-04-16

**Authors:** Di Qu, Panpan Bo, Liankui Wen, Yinshi Sun

**Affiliations:** 1 Institute of Special Animal and Plant Sciences, Chinese Academy of Agricultural Sciences, Changchun 130112, China; qudi2022@163.com (D.Q.); bopanpan89@163.com (P.B.); 2 Institute of Food Science and Engineering, Jilin Agricultural University, Changchun 130118, China; 3 Institute of Chinese Medicinal Materials, Jilin Agricultural University, Changchun 130118, China

**Keywords:** *Panax ginseng*, beverages, chemical composition, PCA, sediment amount prediction

## Abstract

*Panax ginseng* beverages have been some of the most popular plant drinks among consumers in recent years, but they become turbid and sediment are easily formed during production and marketing, these are some of the key issues that affect the quality of the beverages. In this study, we analysed the physicochemical properties of sediments in aqueous extracts of 3- to 6-year-old ginseng, and by tracing the sediment formation process from 0–40 days, we observed that the sediment was gradually beginning on day 10. The solid content of ginseng aged 5 and 6 years was significantly higher than that of ginseng aged 3 and 4 years. There was no significant difference in the sediment amount sediment in the extracts of ginseng of different ages. The light transmittance of the extracts after centrifugation was significantly higher than before centrifugation. Colour-difference analysis found that there was a significant positive correlation between ginseng age and colour-difference value (ΔE). Chemical composition analysis showed that total sugar and proteins were the main components of the sediment. In addition, ginsenosides, amino acids and minerals were also involved in sediment formation to different degrees. A stepwise regression model was established through principal component analysis (PCA), and the regression equation for predicting the sediment amount was obtained as follows: sediment amount (mg/mL) = 2.906 − 0.126 × *C*_Total saponins_ − 0.131 × *C*_Free amino acid__s_.

## 1. Introduction

Ginseng (*Panax ginseng* C. A. Meyer) is a perennial herb of the genus Ginseng in the family Cinchona; it prefers shade, usually flowers after 3 years and bears fruit in years 5–6, and is native to northeastern China, North Korea, South Korea, Japan, and eastern Russia [1,2]. Ginseng is a valuable traditional Chinese medicine that has various physiological effects [3,4,5,6,7], such as antitumor [8,9], antiaging [10,11], antifatigue [12,13], and immune modulation [14,15] effects. Currently, ginseng is widely popular as a food around the world, and 3- to 6-year-old artificially grown ginseng is more commonly available on the market; its growth is greatly influenced by germplasm, soil, age, climate, and water source, which can lead to differences in the chemical composition and pharmacological activity of ginseng [16,17].

Ginseng beverages are plant-based drinks that have been favoured by consumers in recent years, but the production of sediment easily occurs in the production and marketing process; such production is one of the key technical problems hindering the development of ginseng beverages [18]. The formation of sediment is the result of the interaction of various chemical components [19,20]. In the initial stage of storage, small colloidal particles are suspended in the beverage in a cloudy state through Brownian motion. With the extension of storage time, the colloidal particles gradually aggregate into macromolecular particles and precipitate [21]. The common chemical components of ginseng include polysaccharides, proteins, saponins, amino acids, minerals, organic acids, and volatile oil, etc. [22,23,24]. They play different roles in the sediment, thus leading to differences in sediment formation. Studies showed that the chemical composition of ginseng of different ages was different, and the contents of ginsenosides, total saponins, proteins, amino acids, and other nutrients of 3–6-year-old ginseng were the highest [25]. The contents of Re, Rc, Rg_1_, Rg_3_ and Rf contents of ginsenosides at 2–4 years increased with increasing ginseng age, while the content of Rb_1_ peaked at 3 years [26]. Early research has shown that the 30 days prior to storage is the critical period for sediment formation. Ginseng extract sediment has flocculent and granular forms, and sediment can be reversible and irreversible. The floc, mostly reversible sediment, is a polysaccharide–protein flocculate, and they combine via covalent and noncovalent bonds. The complex formed by a covalent bond is irreversible due to strong interactions, while the complex formed by a noncovalent bond can be redissolved by heating and oscillation in the initial stage of sediment formation due to weak forces [27]. The main components that affect the turbidity of ginseng extract are free amino acids, total saponins, Al, Ba, Ca, Fe, Mn, Ni, and Sr. Protein, ginsenosides Rb_1_, Rb_2_, Rb_3_, Rf, elements Al, Fe, Ca, and Na are more likely to participate in the formation of sediment [28]. Therefore, further exploration of the differences in sediment formation of ginseng extracts of different ages is of great significance for the future production of ginseng beverages and accurately predictions of the amount of sediment.

In this study, the sediment of ginseng extracts of 3–6 years of age was observed for 0–40 days, and the differences in the physical properties of the extracts of ginseng of different ages, such as sediment amount, light transmittance, and colour difference were compared. Second, the differences in the chemical components of extracts and sediment of different ages were analyzed, and the key chemical components involved in sediment formation were identified by principal component analysis. On this basis, a stepwise regression analysis model was established to determine the linear relationship between sediment amount and chemical composition, and the sediment amount was predicted based on this relationship.

## 2. Materials and Methods

### 2.1. Materials

White ginseng (3, 4, 5, and 6 years old) samples were obtained from the same planting base in Fusong County, Jilin Province, China.

### 2.2. Preparation of Ginseng Extracts

Ginseng of different ages was crushed and screened for 20–60 mesh particles, extracted with distilled water at a solid–liquid ratio of 1:10 (g/mL) for 60 min at 80 °C, cooled to room temperature, filtered through a double-layer 300 mesh filter cloth, and then centrifuged (5000× *g*, 15 min). The extracts discarded from the bottom sediment were divided into 50 mL centrifuge tubes, pasteurized in a water bath (90 °C, 5 min), and stored at a low temperature (4 °C).

### 2.3. Sediment Formation Observations

The observation team consisted of nine faculty members in food science and a description was made of the different ginseng age extracts during storage based on the sedimentation volume and turbidity.

### 2.4. Preparation of Sediment and Determination of Solids

The extracts stored for 40 days were subjected to high-speed centrifugation (10,000× *g*, 15 min), and the supernatant was decanted while the sediment remained at the bottom. After drying at 80 °C for 48 h, the samples were weighed to determine the amount of sediment present. A total of 20 mL of ginseng extract was dried at 80 °C for 48 h, and the solid weight was determined according to the method described by Nagalakshmi et al. [29].

### 2.5. Measurement of Clarity

The aqueous extracts were centrifuged at 10,000× *g* for 15 min, and the supernatant was collected for testing. Using a 752N Ultraviolet–Visible Spectrophotometer (UV–Vis) (INESA Analytical Instrument Co., Ltd, Shanghai, China) with distilled water as the reference, we measured the transmittance of the supernatant and homogeneous aqueous extracts at 640 nm. The liquid clarity was directly proportional to the transmittance.

### 2.6. Chromatic Aberration Analysis

The chromaticity and colour difference were measured by an NH310 colorimeter (3NH Technology Co., Ltd., Shenzhen, China). Distilled water is used as a reference (L = 32.1, a = −0.65, b = 1.25). where L represents brightness (+) and darkness (−), a represents red (+) and green (−) chromaticity, b represents yellow (+) and blue (−) chromaticity and ΔE represents the combined colour difference.

### 2.7. Chemical Composition Analysis

#### 2.7.1. Free Amino Acid and Protein Analysis

Free amino acids were determined using glutamate as the standard, and the optical density (OD_570_) was measured using an EPOCH enzyme standard (Bio Tek Instruments, Inc., Highland Park, Winooski, USA). The regression equation for free amino acids was *C* = 2.6552 × OD_570_ − 0.1521, *R*^2^ = 0.9946, where *C* is the concentration of free amino acids in mg/mL, and OD_570_ is the OD value at 570 nm. The protein content was determined with a BCA protein assay kit (Sangon Biotechnology, Shanghai, China).

#### 2.7.2. Total Sugar Analysis

Total sugars were determined by the phenol–sulfuric-acid reaction using glucose as a standard. A 2 mL sample was reacted with 1 mL of 5% phenol and 5 mL of sulfuric acid at 40 °C for 30 min and then cooled rapidly to room temperature. The optical density (OD_490_) was then measured using an EPOCH enzyme standard. The regression equation for total sugars was *C* = 8.4567 × OD_490_ + 0.0044, *R*^2^ = 0.9988, where *C* is the concentration of total sugars in mg/mL and OD_490_ is the OD value at 490 nm.

#### 2.7.3. Total Saponin Analysis

The total saponin content was determined by the vanillin–sulfuric-acid method using ginsenoside Re (Shanghai Yuanye Biotechnology Co., Ltd., Shanghai, China) as a standard. A 40 μL sample was swept dry, and 0.2 mL of 5% vanillin (5 g vanillin dissolved in 100 mL of glacial acetic acid) and 1 mL of 72% sulfuric acid were added at 60 °C for 15 min. After cooling, 5 mL of glacial acetic acid was added, and the solution was mixed thoroughly. The optical density (OD_560_) was measured using an EPOCH microplate reader. The regression equation for total saponins was *C* = 1.2397 × OD_560_ + 0.0045, *R*^2^ = 0.9955, where *C* is the concentration of total saponins in mg/mL, and OD_560_ is the OD value at 560 nm.

#### 2.7.4. Mineral Analysis

The mineral elemental composition was determined using an inductively coupled plasma optical emission spectrometer (ICP-OES) (Varian 710-ES, Palo Alto, America). First, 0.5 mL of sample and 0.1 g of solid sample were dissolved in an acidic mixture of 2.5 mL HClO_4_ and 10 mL HNO_3_ and diluted to 100 mL using distilled water.

#### 2.7.5. Analysis of Individual Ginsenosides

The Rg_1_, Re, Rf, Rb_1_, Rc, Rb_2_, Rb_3_, and Rd ginsenoside standards (Shanghai Yuanye Biotechnology Co., LTD, Shanghai, China) were accurately weighed and dissolved in methanol at a concentration of 0.5 mg/mL. The 8 standard solutions were dissolved in 10 mL of methanol to prepare a mixed standard solution and diluted to different concentrations to establish a standard curve for standby. The mixed standards and samples were filtered through a 0.22 μm aqueous filter membrane and placed on the machine to be measured.

Analysis was performed using ultra-performance liquid chromatography (UPLC) (Waters ACQUITY UPLC, America). An Acquity UPLC H-Class C18 column (2.1 mm × 50 mm, 1.7 μm, Waters, Ireland) was used. The mobile phase consisted of solvent A (purified water) and solvent B (acetonitrile) with a gradient elution program of 0–5.8 min (13–22% B); 5.8–18.75 min (22–38% B); 18.85–22.05 min (38–40% B); 22.05–23.55 min (40–45% B); 23.55–24.25 min (45–58% B); 24.25–26.75 min (58–62% B); 26.75–30.75 min (62–80% B); 30.75–37.25 min (80–100% B) and 37.25–40 min (100–13% B) with a flow rate of 0.4 mL/min and a sample volume of 3 μL. The chromatograms were detected at 203 nm.

The regression equations for the ginsenosides were as follows: Rg_1_: *Y =* 2,340,000x + 7480, *R*^2^ = 0.9991; Re: *Y* = 1,960,000x + 7420, *R*^2^ = 0.9992; Rf: *Y* = 2,390,000x + 1690, *R*^2^ = 0.9987; Rb_1_: *Y =* 1,280,000x + 1430, *R*^2^ = 0.9990; Rc: *Y* = 1,300,000x + 1250, *R*^2^ = 0.9987; Rb_2_: *Y =* 1,360,000x + 1480, *R*^2^ = 0.9989; Rb_3_: *Y* = 1,300,000x + 1250, *R*^2^ = 0.9988; Rd: *Y* = 1,320,000x + 1120, *R*^2^ = 0.9970, where *Y* represents the peak area, and x represents the content of ginsenosides.

### 2.8. Statistical Analysis

The chemical composition of the different ginseng age extracts was expressed as the mean ± SD (*n* = 3). One-way ANOVA, significant difference analysis, principal component analysis (PCA), and stepwise regression analysis were performed using SPSS (version 18, SPSS Inc., Chicago, IL, USA).

## 3. Results and Discussion

### 3.1. Sediment Formation of Ginseng Extract at Different Ages

**Figure 1:**
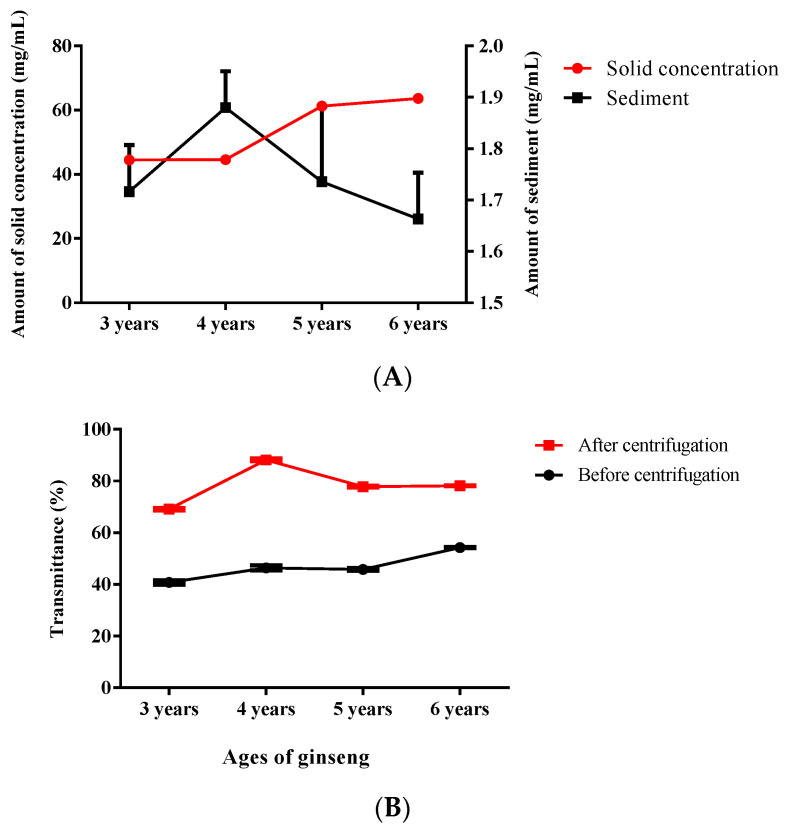
(**A**): Solid concentration and amount of sediment in ginseng extract of different ages, (**B**): the transmittance of ginseng extract of different ages before and after centrifugation. The data shown are the means of three replicates (*n* = 3).

With the extension of the storage time, the sediment amount in each ginseng age extract gradually increased (Supplementary Material Table S1). No sediment formed in any of the samples on Day 0, and significant sediment began to appear on Day 10, with significantly more sediment on Day 40 than on Day 10. The protein polysaccharides in the ginseng extracts produced small colloidal particles suspended in the liquid in a turbid state at the beginning of storage through electrostatic interactions and gradually sedimented to the bottom as the small colloidal particles gathered and formed large particles over time [30,31]. The three-year-old ginseng extracts were turbid, and the difference in the turbidity characteristics might have been due to the different contents of turbid active proteins [32]. According to measurements of the solids and the amount of sediment (Figure 1A), the solid content increased with the age of the ginseng, but the differences were not significant at 3 and 4 years or at 5 and 6 years. The change in the amount of sediment with growth years was different from the trend of the solids over the growth years; the results were as follows: 4 > 5 > 3 > 6, and the sediment amount was 3.9%, 4.2%, 2.8%, and 2.6% of the solids, respectively. It was obvious that 3- and 4-year-old ginseng were more likely to form sediment. The light transmission of the extract before centrifugation tended to increase with the age of the ginseng (Figure 1B), and the light transmission of the sample of 4-year-old ginseng after centrifugation was significantly higher than the ginseng of other ages, which indicates that the main component of the 4-year-old ginseng extract that causes turbidity in the liquid is involved in the highest degree of sediment formation.

### 3.2. Analysis of the Chemical Composition

Routine chemical composition analysis: There were some differences in the chemical composition of the supernatant and sediments of ginseng at different ages (Table 1). Free amino acids were the highest in ginseng supernatant at 3 years and the lowest at 4 years. The total sugars and total saponins increased significantly after 4 years, and there was no significant difference in protein. Free amino acids, proteins, and total saponins in the sediment tended to decrease with the increase in ginseng age. Total sugars accounted for the largest proportion of the 4 conventional chemical components in the sediment, followed by proteins, and the total saponins of 3-year-old ginseng formed a significantly greater portion of the sediment than in the ginseng of other ages. It has been shown that soluble proteins decrease and protein polymers gradually form in heat-treated samples as storage time increases, and that there is an important relationship between protein aggregation and storage time [33]; given enough time, it is possible for protein polymers that are large enough to precipitate and form in the sample [34,35]. In addition, polysaccharides bind to proteins electrostatically to produce insoluble polymers [36], and anionic polysaccharides such as pectin can bind to cationic substances, such as Ca^2+^, to form insoluble substances [37,38,39,40].

Analysis of ginsenosides: Ginsenosides are important active substances in ginseng and have different biological activities. Eight ginsenosides were analysed in the extracts and sediments of ginseng of different ages. There were significant differences in the content of ginsenosides in the supernatant with the change in ginseng age, and the total amount of ginsenosides differed as follows: 4 > 6 > 5 > 3 years; each ginsenoside was involved in the formation of sediments to different degrees. According to the content, Rf and Rb_3_ accounted for the smallest proportion of the sediment, and the total amount of ginsenosides accounted for 6.9%, 6.7%, 4.1%, and 4.7% of the sediment for each ginseng age, respectively. They accounted for a significantly higher portion of the sediments in the 3- and 4-year-old ginseng than in the 5- and 6-year-old ginseng, which indicates that the older the ginseng age is, the lower the degree of participation of total ginsenosides in the sediment. The main reason for the difference could be that the Re, Rc, and Rd contents decreased significantly with increasing ginseng age.

Mineral analysis: The contents of Mg, Na, Sr, Zn and total minerals in the supernatant decreased significantly after 3 years of ginseng age; the contents of Mn, Ni, and K showed an increasing trend with the increase in ginseng age; there was no significant difference in Al, Ba, and Fe; the contents of Ca and Cu in 4 years of ginseng were significantly lower than other ginseng ages; all minerals in the sediment except Al and Ni showed a decreasing trend with the increase in ginseng age, and all of them were within 3 years (Table 2). The contents of Ca, Mg, Na, and K in the ginseng extracts and sediments of the four ginseng ages were relatively high. It has been shown that metal ions are one of the key factors that cause turbidity and sediment in beverages, especially Ca^2+^, Mg^2+^, Fe^2+^, Al^3+^, and other metal ions [41]. During beverage processing, they easily combine with polyphenols, organic acids, and other chemical components to cause beverage turbidity and sediment. They can also combine with polysaccharides, forming a stable “egg-box” structure; for example, Ca^2+^ and pectin can combine [42]. Deionized or distilled water should be used in the production of beverages to reduce the production of turbidity and sediment [43].

### 3.3. Colour Difference Analysis

The colour differences of aqueous extracts of 3- to 6-year-old ginseng were analysed (Table 3). The lightness and darkness L decreased significantly with increasing ginseng age, and there was no significant difference between 5 and 6 years of age. The 3-year-old ginseng extract was greenish, and the redness of the 4-, 5- and 6-year-old ginseng extracts became significantly darker with increasing ginseng age. The yellowness gradually became darker with increasing ginseng age, and the comprehensive colour difference (ΔE) values of ginseng of different ages were significantly different. The colour difference was more obvious with increasing ginseng age. The browning of the extract after heating is closely related to the Maillard reaction. The reason for the darkening of the colour may be that the total sugar content in ginseng increases significantly with increasing ginseng age, and the degree of carbonyl ammonia reaction is greater. In addition, the colour difference may also be caused by the different contents of pigment substances in ginseng of different ages [44,45].

### 3.4. Analysis of Key Chemical Components

To predict the amounts of sediment in ginseng extracts in advance, PCA was performed on 24 chemical components of ginseng extracts to obtain the main chemical indicators that could predict the sediment, and the results showed that the cumulative contribution of the first four chemical components was 91.00%. According to the absolute coefficients of each chemical component, the first principal component was represented by free amino acids (coefficient 0.912), the second was total sugars (coefficient 0.980), the third was Al (coefficient 0.765), and the fourth was total saponins (coefficient 0.392) (Table 4). Based on these four principal components, stepwise regression analysis was performed, and it was concluded that total saponins and free amino acids were the key factors that affected the sediment amount. The regression relationship equation was as follows: sediment amount (mg/mL) = 2.906 − 0.126 × *C*_Total saponins_ − 0.131 × *C*_Free amino acid__s_, where *C*_Total saponins_ was the amount of total saponins in the extract, and *C*_Free amino acid__s_ was the free amino acid content in the extract (Table 5), which indicates that the model had a good predictive effect. Both the total saponin and free amino acids contents in the extracts were negatively correlated with the sediment amount.

## 4. Conclusions

The results of the study showed that the sediment was gradually deposited from day 10. There were differences in the chemical composition of the ginseng extract and its sediment of different ginseng ages. Polysaccharides and proteins were the main components of the sediment, and ginsenosides, amino acids, and minerals also participated in the formation of the sediment to varying degrees. The solid content of the high-age (5, 6 years) ginseng extracts was significantly higher than that of the low-age (3, 4 years) extracts. The colour difference (ΔE), a, and b of the ginseng extract increased with increasing ginseng age, and the lightness L decreased with increasing ginseng age. PCA showed that free amino acids, total sugars, Al, and total saponins were the main chemical factors for the formation of sediment. Further stepwise regression analysis established the relationship equation for predicting sediment: sediment amount (mg/mL) = 2.906 − 0.126 × *C*_Total saponins_ − 0.131 × *C*_Free amino acid__s_.

## Figures and Tables

**Table 1:** Analysis of the chemical composition of the extract and sediment of ginseng of different ages.

Chemical Components (mg/mL)	Supernatant				Sediment			
	3 Years	4 Years	5 Years	6 Years	3 Years	4 Years	5 Years	6 Years
Free amino acid	4.88 ± 0.17 b	3.93 ± 0.10 a	4.54 ± 0.16 b	4.12 ± 0.34 a	0.24 ± 0.01 b	0.23 ± 0.03 b	0.21 ± 0.01 ab	0.19 ± 0.01 a
Protein	3.30 ± 0.48 a	3.14 ± 0.41 a	3.61 ± 0.25 a	3.37 ± 0.14 a	0.82 ± 0.03 b	0.81 ± 0.01 b	0.61 ± 0.11 a	0.51 ± 0.09 a
Total sugar	32.47 ± 4.95 a	29.16 ± 2.83 a	53.13 ± 1.78 b	54.51 ± 3.31 b	2.18 ± 0.06 b	1.95 ± 0.11 a	2.18 ± 0.21 ab	2.26 ± 0.05 b
Total saponins	4.25 ± 0.10 a	4.043 ± 0.14 a	4.783 ± 0.55 ab	5.542 ± 0.66 b	1.04 ± 0.33 b	0.121 ± 0.11 a	0 a	0.05 ± 0.08 a
Rg1	0.16 ± 0.00 a	0.31 ± 0.00 c	0.29 ± 0.00 b	0.33 ± 0.01 d	0.01 ± 0.00 a	0.02 ± 0.00 b	0.01 ± 0.00 a	0.01 ± 0.00 a
Re	0.28 ± 0.00 c	0.31 ± 0.00 d	0.19 ± 0.00 a	0.23 ± 0.00 b	0.03 ± 0.00 c	0.02 ± 0.00 b	0.01 ± 0.00 a	0.01 ± 0.00 a
Rf	0.04 ± 0.00 a	0.10 ± 0.00 d	0.07 ± 0.00 b	0.09 ± 0.01 c	0 a	0.01 ± 0.00 b	0 a	0.01 ± 0.00 b
Rb1	0.23 ± 0.01 a	0.47 ± 0.01 c	0.36 ± 0.00 b	0.51 ± 0.01 d	0.02 ± 0.00 a	0.03 ± 0.01 b	0.02 ± 0.00 a	0.03 ± 0.00 b
Rc	0.12 ± 0.03 a	0.21 ± 0.02 bc	0.18 ± 0.02 b	0.23 ± 0.01 c	0.03 ± 0.00 b	0.01 ± 0.00 a	0.01 ± 0.00 a	0.01 ± 0.00 a
Rb2	0.13 ± 0.01 a	0.27 ± 0.01 d	0.15 ± 0.00 b	0.19 ± 0.00 c	0.01 ± 0.00 a	0.02 ± 0.00 b	0.01 ± 0.00 a	0.01 ± 0.00 a
Rb3	0.01 ± 0.00 a	0.03 ± 0.00 c	0.02 ± 0.00 b	0.02 ± 0.00 b	0 a	0 a	0 a	0 a
Rd	0.13 ± 0.02 c	0.31 ± 0.01 d	0.07 ± 0.00 a	0.12 ± 0.00 b	0.02 ± 0.00 b	0.03 ± 0.00 c	0 a	0 a
Total ginsenoside	1.10 ± 0.04 a	2.01 ± 0.03 d	1.33 ± 0.04 b	1.71 ± 0.04 c	0.12 ± 0.01 b	0.13 ± 0.02 b	0.07 ± 0.01 a	0.08 ± 0.00 a

Note: The data shown are the means ± SD (*n* = 3), and different letters labeled with the same row of data represent significant differences. (*p* < 0.05).

**Table 2:** Analysis of the mineral elements of the extract and sediment of ginseng of different ages.

Mineral Elements (μg/mL)	Supernatant				Sediment			
	3 Years	4 Years	5 Years	6 Years	3 Years	4 Years	5 Years	6 Years
Al	0.92 ± 0.23 a	1.00 ± 0.24 a	0.76 ± 0.10 a	1.23 ± 0.22 a	0.72 ± 0.23 a	0.91 ± 0.27 a	0.95 ± 0.20 a	0.70 ± 0.11 a
Ba	0.23 ± 0.08 a	0.16 ± 0.01 a	0.20 ± 0.01 a	0.15 ± 0.01 a	0.14 ± 0.03 b	0.03 ± 0.03 a	0.04 ± 0.01 a	0.02 ± 0.00 a
Ca	49.60 ± 6.53 b	35.78 ± 0.75 a	43.49 ± 1.74 ab	42.13 ± 1.04 ab	28.98 ± 8.70 b	12.68 ± 1.62 a	11.14 ± 2.95 a	11.04 ± 0.70 a
Cu	0.43 ± 0.06 b	0.12 ± 0.01 a	0.44 ± 0.08 b	0.40 ± 0.07 b	0.41 ± 0.04 b	0.06 ± 0.02 a	0.06 ± 0.01 a	0.08 ± 0.05 a
Fe	0.93 ± 0.31 a	1.24 ± 0.11 a	1.02 ± 0.13 a	1.33 ± 0.15 a	0.48 ± 0.11 c	0.27 ± 0.15 bc	0.228 ± 0.060 a	0.22 ± 0.02 a
Mg	106.67 ± 4.96 b	80.50 ± 0.75 a	76.34 ± 0.57 a	78.22 ± 1.61 a	49.30 ± 7.92 b	6.02 ± 1.50 a	4.15 ± 1.08 a	4.43 ± 0.22 a
Mn	1.72 ± 0.22 ab	2.43 ± 0.03 b	2.70 ± 0.03 b	3.88 ± 0.04 c	1.42 ± 0.38 b	0.06 ± 0.04 a	0.15 ± 0.03 a	0.22 ± 0.02 a
Na	25.88 ± 1.84 c	16.17 ± 0.25 a	18.36 ± 0.57 ab	19.98 ± 1.76 b	19.88 ± 1.33 b	14.17 ± 3.15 a	10.90 ± 0.93 a	9.82 ± 1.03 a
Ni	0.07 ± 0.01 b	0.03 ± 0.01 a	0.09 ± 0.01 bc	0.11 ± 0.02 c	0.01 ± 0.02 a	0.02 ± 0.01 a	0.02 ± 0.00 a	0.02 ± 0.01 a
Sr	0.37 ± 0.06 c	0.26 ± 0.00 a	0.30 ± 0.00 bc	0.27 ± 0.01 b	0.17 ± 0.06 b	0.06 ± 0.01 a	0.05 ± 0.01 a	0.05 ± 0.01 a
Zn	1.32 ± 0.19 b	0.72 ± 0.04 a	0.67 ± 0.03 a	0.86 ± 0.12 a	0.45 ± 0.27 b	0.05 ± 0.03 a	0.04 ± 0.022 a	0.06 ± 0.02 a
K	556.14 ± 12.16 b	505.22 ± 1.62 b	470.83 ± 14.70 b	466.86 ± 25.94 a	60.67 ± 3.12 b	54.21 ± 7.48 a	30.40 ± 7.31 a	32.34 ± 3.37 a
Total	744.28 ± 26.65 c	643.61 ± 3.81 b	615.19 ± 17.96 a	615.40 ± 30.99 a	162.63 ± 22.19 c	88.54 ± 14.29 b	58.08 ± 12.61 a	58.99 ± 5.55 a

Note: The data shown are the means ± SD (*n* = 3), and different letters labeled with the same row of data represent significant differences. (*p* < 0.05).

**Table 3:** Colorimetric parameters of ginseng extract of different ages.

Age (Year)	L	a	b	ΔE
3	31.63 ± 0.02 c	−0.25 ± 0.00 a	0.49 ± 0.01 a	0.98 ± 0.00 a
4	26.8 ± 0.01 b	2.77 ± 0.01 b	3.31 ± 0.01 b	6.64 ± 0.02 b
5	23.26 ± 0.00 a	5.47 ± 0.02 c	4.31 ± 0.00 c	11.18 ± 0.01 c
6	23.25 ± 0.02 a	5.66 ± 0.02 d	4.96 ± 0.01 d	11.25 ± 0.00 d

Note: The data shown are the means ± SD (*n* = 3), and different letters labeled with the same column of data represent significant differences. (*p* < 0.05).

**Table 4:** PCA of the chemical components of ginseng extracts of different ages.

Chemical Components	Component			
	1	2	3	4
Free amino acid	0.912	0.131	−0.069	−0.007
Protein	0.465	0.230	−0.119	−0.019
Total sugar	−0.107	0.980	−0.007	−0.034
Total saponins	−0.122	0.834	0.235	0.392
Rg1	−0.937	0.334	0.024	−0.080
Re	−0.057	−0.924	0.327	0.176
Rf	−0.973	−0.015	0.113	−0.007
Rb1	−0.931	0.236	0.242	0.078
Rc	−0.853	0.322	0.264	−0.128
Rb2	−0.853	−0.459	0.217	−0.034
Rb3	−0.901	−0.306	0.255	−0.025
Rd	−0.550	−0.804	0.183	0.028
Total ginsenoside	−0.938	−0.229	0.249	0.016
Al	−0.169	0.167	0.765	0.332
Ba	0.661	−0.044	0.360	−0.605
Ca	0.842	0.261	0.379	−0.243
Cu	0.632	0.623	−0.162	0.093
Fe	−0.485	0.164	0.711	−0.271
Mg	0.841	−0.424	0.296	0.091
Mn	0.132	0.570	0.759	0.035
Na	0.884	0.013	0.280	0.298
Ni	0.234	0.906	0.097	0.085
Sr	0.852	−0.015	0.389	−0.316
Zn	0.799	−0.189	0.502	0.163
K	0.609	−0.679	0.093	0.073
Total minerals	0.792	−0.561	0.136	0.166
Cumulative variance (%)	49.82	74.84	86.78	91.00

**Table 5:** Results of multiple stepwise regression analysis.

Model	*R*	*R* ^2^		Unstandardized Coefficients		Standardized Coefficients	t	Sig
				B	SE	β		
1	0.754	0.568	(constant)	2.352	0.169		13.985	0.000
			Total saponins	−0.13	0.36	−754	−3.628	0.005
2	0.882	0.777	(constant)	2.906	0.229		12.686	0.000
			Total saponins	−0.126	0.027	−0.731	−4.643	0.001
			Free amino acid	−0.131	0.045	−0.458	−2.908	0.017

Note: Stepwise regression model (*F* ≤ 0.05 selected, *F* ≥ 0.10 rejected), with the dependent variable being the amount of ginseng extract sedimented.

## Supplementary Materials

The following supporting information can be downloaded at: https://www.mdpi.com/article/10.3390/foods11081161/s1, Table S1: Observation of sediment formation of ginseng extract of different ages; Table S2: PUBCHEM CID number for ginsenoides in this article.
