# Supplementary material for: Analysis of Key Chemical Components in Aqueous Extract Sediments of Panax Ginseng at Different Ages
Source: Foods. 2022 Apr 16;11(8):1161. doi: 10.3390/foods11081161 (PMC9025099; doi:10.3390/foods11081161)
Supplement: Supplementary file 1 [file foods-11-01161-s001.zip › foods-1651777-supplementary.pdf]

Supplementary materials

Table S1. Observation of sediment formation of ginseng extract of different ages

Time (day)	3years	4years	5years	6years
0	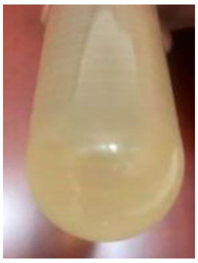<br>Slight turbidity, no sediment	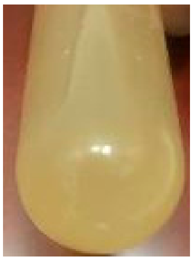<br>Turbidity, no sediment	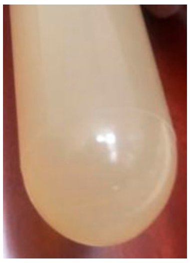<br>Turbidity, no sediment	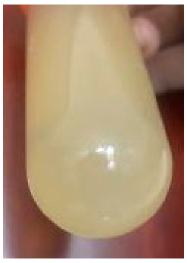<br>Turbidity, no sediment
10	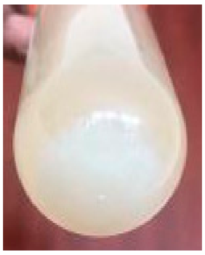<br>Turbidity, little sediment	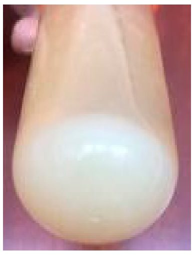<br>Slight turbidity, much sediment	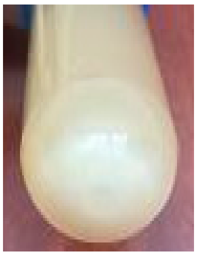<br>Turbidity, little sediment	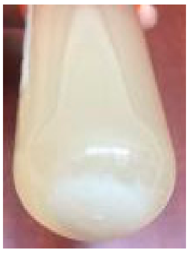<br>Turbidity, little sediment
20	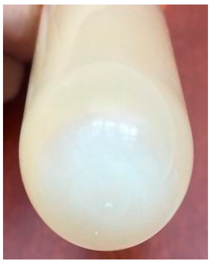<br>Turbidity, much sediment	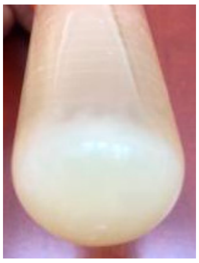<br>Slight turbidity, much sediment	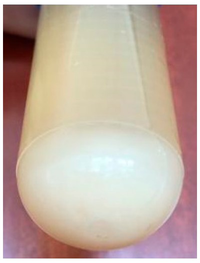<br>Slight turbidity, much sediment	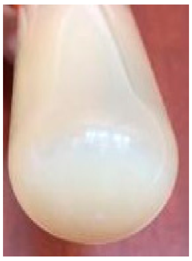<br>Turbidity, much sediment
40	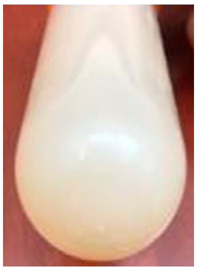<br>Turbidity, large sediment	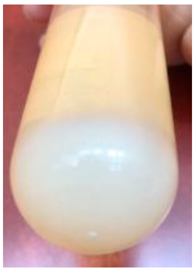<br>Limpid, large sediment	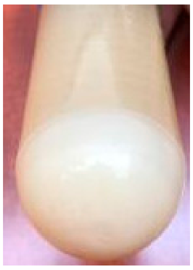<br>Slight turbidity, large sediment	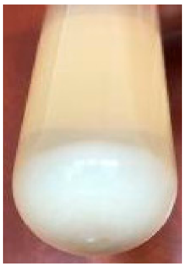<br>Limpid, large sediment

Table S2. PUBCHEM CID number for ginsenoides in this article

Ginsenosides	PUBCHEM CID
Rg_1	441923
Re	441921
Rf	441922
Rb_1	9898279
Rc	12855889
Rb_2	6917976
Rb_3	12912363
Rd	24721561
